# RNA-Dependent Oligomerization of APOBEC3G Is Required for Restriction of HIV-1

**DOI:** 10.1371/journal.ppat.1000330

**Published:** 2009-03-06

**Authors:** Hendrik Huthoff, Flavia Autore, Sarah Gallois-Montbrun, Franca Fraternali, Michael H. Malim

**Affiliations:** 1 Department of Infectious Diseases, King's College London, London, United Kingdom; 2 Randall Division of Cell and Molecular Biophysics, King's College London, London, United Kingdom; Northwestern University, United States of America

## Abstract

The human cytidine deaminase APOBEC3G (A3G) is a potent inhibitor of retroviruses and transposable elements and is able to deaminate cytidines to uridines in single-stranded DNA replication intermediates. A3G contains two canonical cytidine deaminase domains (CDAs), of which only the C-terminal one is known to mediate cytidine deamination. By exploiting the crystal structure of the related tetrameric APOBEC2 (A2) protein, we identified residues within A3G that have the potential to mediate oligomerization of the protein. Using yeast two-hybrid assays, co-immunoprecipitation, and chemical crosslinking, we show that tyrosine-124 and tryptophan-127 within the enzymatically inactive N-terminal CDA domain mediate A3G oligomerization, and this coincides with packaging into HIV-1 virions. In addition to the importance of specific residues in A3G, oligomerization is also shown to be RNA-dependent. Homology modelling of A3G onto the A2 template structure indicates an accumulation of positive charge in a pocket formed by a putative dimer interface. Substitution of arginine residues at positions 24, 30, and 136 within this pocket resulted in reduced virus inhibition, virion packaging, and oligomerization. Consistent with RNA serving a central role in all these activities, the oligomerization-deficient A3G proteins associated less efficiently with several cellular RNA molecules. Accordingly, we propose that occupation of the positively charged pocket by RNA promotes A3G oligomerization, packaging into virions and antiviral function.

## Author Summary

APOBEC3G is a human protein that inhibits the replication of HIV-1 in CD4+ T cells. It gains entry to the virus particles that are released from infected cells and subsequently interferes with viral genome replication, which in the case of HIV-1 is reverse transcription. APOBEC3G is a cytidine deaminase, and it catalyses the deamination of cytidines to uridines in viral single-stranded DNA replication intermediates, resulting in the generation of defective progeny viruses. In addition, APOBEC3G can inhibit reverse transcription by a poorly characterized deamination-independent mechanism. HIV-1 has evolved the viral Vif protein to counteract the antiviral properties of APOBEC3G. Vif associates with APOBEC3G and targets it for proteasomal degradation, such that intracellular levels of APOBEC3G are reduced and packaging into virions is averted. Based on the structure of a human homolog of APOBEC3G, APOBEC2, we performed a mutational analysis of amino acids that have the potential to mediate the assembly of APOBEC3G into multi-component complexes. We report that these amino acids affect the association of APOBEC3G with itself and cellular RNA, and that the same attributes are also required for packaging into virions and antiviral function. Thus, the processes of APOBEC3G self-association, RNA binding, and virion packaging are functionally linked and essential for virus inhibition.

## Introduction

The human protein APOBEC3G (A3G) belongs to a family of cellular polynucleotide cytidine deaminases and is a potent inhibitor of HIV-1 in the absence of the viral protein Vif [1]. Vif-deficient HIV-1 (HIV-1/Δ*vif*) is subject to A3G mediated cytidine to uridine deamination of single-stranded DNA that is generated during reverse transcription, a process also referred to as DNA editing or hypermutation [2]–[4]. In addition, A3G further suppresses infection by inhibiting reverse transcription in a poorly understood manner that seems to be independent of the deamination activity of the protein [5]–[9]. A3G is incorporated into progeny virions during particle assembly at the plasma membrane by associating with the NC domain of the viral Gag protein in an RNA dependent manner [10]–[15]. The viral Vif protein prevents the antiviral properties of A3G by targeting it for proteasomal degradation [16]. Specifically, Vif interacts with A3G and recruits the cullin5-elonginB/C-Rbx ubiquitin ligase complex, resulting in the polyubiquitylation and degradation of A3G [17]. This reduction of intracellular levels in A3G results in a substantial decrease in the packaging of A3G into virus particles and, therefore, suppresses its antiviral properties.

The recent reports of the crystal structure of APOBEC2 (A2) [18] and the NMR and crystal structures of the C-terminal cytidine deaminase (CDA) domain of A3G [19],[20] offer opportunities to investigate the structure-function organization of APOBEC proteins with greater incisiveness. Although the physiological function of A2 is as yet unknown, its structure shows all the hallmarks of a cytidine deaminase, being a five-stranded mixed β-sheet which presents on one face two α-helices containing the H/C-X-E-X_23–28_-P-C-X_2_-C catalytic centre that coordinates a zinc ion. Surprisingly, A2 associates into tetramers in a manner unprecedented among cytidine deaminases. Whereas tetrameric free-nucleotide cytidine deaminases of bacteria [21],[22], yeast [23] and vertebrates [24] adopt a globular structure in which each subunit interacts with the other three, A2 forms a linear tetramer in which monomers interact with either one or two of the other subunits [18]. An A2 monomer contains a single CDA domain and two of these form a dimer by joining the β-sheets present in each monomer in a side-by-side fashion such that one wide β-sheet is formed. The tetramer is assembled from two such dimers through head-to head interactions at one edge of the extended β-sheets.

In contrast to A2, A3G contains two CDA domains in a single polypeptide chain, which are termed the N- and C-terminal CDA domains (N-CDA and C-CDA, respectively). Indeed, the CDA fold observed in the A2 crystal structure closely matches the structure of the truncated A3G C-CDA domain as observed by NMR and crystallography [19],[20]. Differences arise mainly at the peripheral loops, which are generally longer in A3G than in A2. The A3G C-CDA fragment is exclusively monomeric, both in solution and in crystalline form [19],[20]. However, there is mounting evidence that A3G not only oligomerizes [12], [25]–[30], but can also assemble into large RNP complexes that accumulate in cellular microdomains that are associated with RNA regulation, such as P-bodies and stress granules [30]–[34]. We therefore asked whether the tetrameric structure of A2 may hold clues not only into how A3G oligomerizes, but also into its participation in other interactions. Here, we show that hydrophobic residues in A3G that are equivalent to those that mediate A2 oligomerization are required for RNA-dependent oligomerization, packaging into HIV-1 virions and the inhibition of HIV-1 infection. In addition, we present a homology model of an A3G dimer that reveals a positively charged pocket at the predicted dimer interface. Mutation of basic residues within this pocket also affects oligomerization, RNA interactions, virion packaging and virus inhibition.

## Results

### Differential contributions of A3G N- and C-CDA domains to packaging, DNA editing, and oligomerization

**Figure 1:**
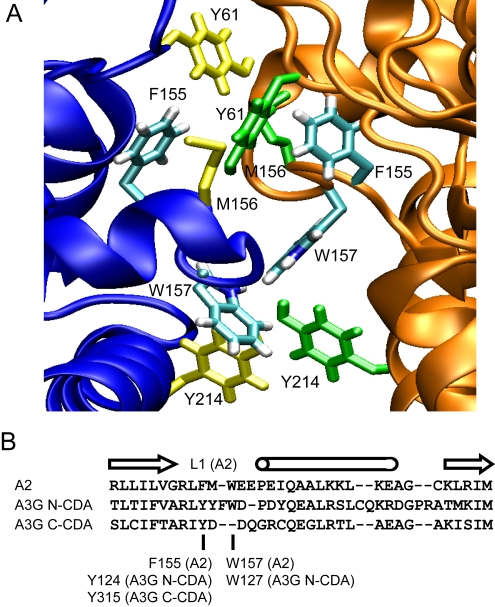
The tetramer interface of A2 and sequence alignment with A3G. (A) Detail of the A2 tetramer interface, highlighting residues that mediate oligomerization interactions. One subunit is shown in blue (left) and one in orange (right). Residues F155 and W157 are shown in blue, other residues from the left-hand subunit that contribute interactions are shown in yellow, and residues from the right-hand subunit are in green; all are indicated by labels. (B) Sequence alignment of A2 with the A3G N- and C-CDA domains corresponding to the L1 loop of A2. Arrows indicate the position of β-strands, and a barrel indicates the position of an α-helix in the A2 crystal structure. Below the alignment, residues in A3G are indicated that correspond to F155 and W157 in A2.

The tetramer interface of A2 is formed of extensive hydrophobic, polar and electrostatic interactions, many of which are clustered in a loop termed L1 [18]. In particular, residues F155, M156 and W157 mediate key hydrophobic interactions (Figure 1A), whereas R153, E158 and E159 are involved in salt-bridges as well as in hydrogen bonding. Upon alignment of the A2 amino acid sequence with the N- or C-terminal CDA sequences of A3G, we identified a highly similar loop sequence in both CDA domains of A3G in which both charged and hydrophobic residues are conserved (Figure 1B). Arginines equivalent to R153 in A2 are present at positions 122 and 313 in A3G, whereas the tyrosines at positions 124 and 315 in A3G are at the equivalent position of F155 in A2. Although F155 does not make any direct interactions across the tetramer interface of the A2 crystal, it is involved in a cluster of hydrophobic packing interactions that sandwich M156 between Y61 and F155 at the tetramer interface. A tryptophan equivalent to W157 in A2 is present only in the N-CDA of A3G at position 127. W157 of A2 makes extensive hydrophobic interactions across the tetramer interface, notably with Y214 and W157 of the adjacent subunit.

We have previously reported a mutational analysis of residues 122–146 of A3G to define the site of interaction with Vif, which was mapped at positions 128–130 [35]. That analysis also revealed that substitutions at positions Y124 and W127 yield A3G proteins that are inefficiently packaged into virus particles and therefore lose their antiviral properties. Given the involvement of the conserved counterparts of these residues in A2 oligomerization, we sought to establish whether this region would have an analogous activity in A3G. To investigate this possibility, and to compare the contribution that the N- or C-CDAs of A3G may make to oligomerization, we introduced identical mutations (alanine, leucine and phenylalanine) at residues Y124 and Y315; these residues were chosen because they are present at equivalent positions in both the N- and C-CDA of A3G, and because the mutant proteins are expressed well. In contrast, the introduction of substitutions at the conserved arginine at position 122 resulted in poor expression [35], and mutants of R122 were therefore not examined further. The construction of mutations at position W127 has been described previously [35].

**Figure 2:**
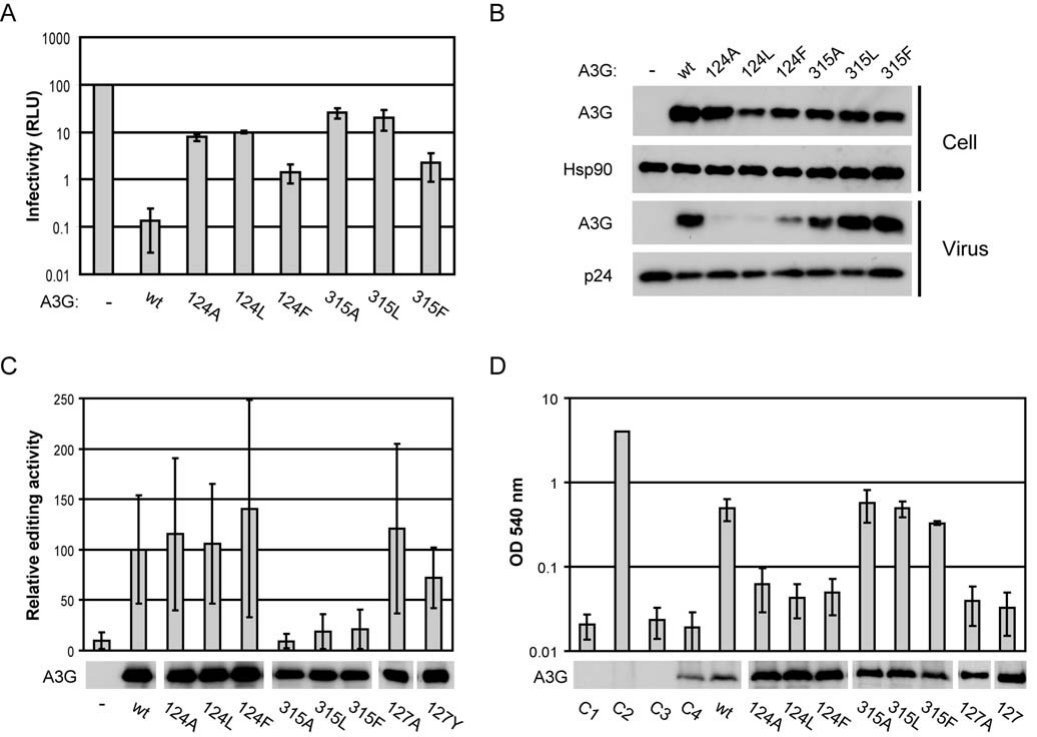
Characterization of A3G proteins with mutations at Y124, W127, and Y315. (A) Single-cycle infectivity of HIV-1/Δ*vif* viruses produced in the presence of wild type or mutant A3G, indicated on the x-axis, as measured in relative luciferase units and presented as percent infectivity relative to the wild type A3G (RLU, y-axis). The empty vector control is indicated by -. The data are the average of three independent experiments, and errors bars represent the standard deviation of the three independent transfections. (B) Expression of wild type and mutant A3G proteins in 293T producer cells and packaging into HIV-1/Δ*vif* virions as determined by immunoblotting. The empty vector control is indicated by -. (C) Relative editing activity of wild type and mutant A3G proteins as determined in a bacterial mutator assay relative to activity of wild type A3G from 12 independent experiments. The empty vector control is indicated by -. Immunoblots beneath the graph show the expression of A3G in equal volumes of the bacterial cultures. (D) Interaction of wild type and mutant A3G proteins (prey) with wild type A3G (bait) in a yeast two-hybrid assay as determined by β-galactosidase activity in OD_540_ units. A series of controls include: C1, empty bait and prey vectors; C2, the positive control with Tsg101-bait and Vps28-prey; C3, wild type A3G-bait and empty prey vector; C4, empty bait vector and wild type A3G-prey. The data are the average of three independent experiments. Beneath the graph, immunoblots using anti-HA antibody show the expression in equal volumes of the yeast cultures of A3G prey, which uniquely carry a triple HA –tag.

We first tested these mutant A3G proteins for their ability to inhibit HIV-1/Δ*vif* infection (Figure 2A). The substitution of Y124 or Y315 to alanine or leucine caused marked losses of antiviral function, whereas substitutions to the chemically more similar phenylalanine resulted in less marked disruption. Determination of the A3G content of virus particles revealed that all mutations at position Y124 result in poor packaging, whereas packaging was maintained with mutations at position Y315 (Figure 2B). Interestingly, the Y124F mutation yielded low but clearly detectable levels of A3G in virions in comparison to mutants Y124A and Y124L, which likely explains why this protein showed a less severe loss of antiviral activity. We next determined the extent to which wild type or mutant A3G can act as a mutagen in a bacterial DNA editing assay (Figure 2C). In this analysis, we included two mutants of W127 (W127A and W127Y), which have previously been shown to have substantial packaging defects [35]. Editing activity was maintained following substitutions at positions Y124 and W127, but mutations at position Y315 caused a loss of editing. Together, these results indicate that the loss of antiviral activity imparted by mutations at residues Y124 and W127 corresponds to reduced packaging, whereas DNA editing activity is unaffected. Conversely, mutations at residue Y315 ablate DNA editing but not packaging into virions, which is consistent with the critical involvement of Y315 in substrate DNA binding at the catalytically active C-CDA domain [19],[20].

To begin to address the ability of A3G to oligomerize, we performed a yeast two-hybrid experiment (Figure 2D). Mutations were introduced into the prey-construct and assayed with a wild type A3G bait. Again, we observed a marked difference between the effects of substitutions in the N- and C-CDA domains of A3G. Mutations at Y124 and W127 resulted in a lack of reporter gene activity, whereas mutations at Y315 displayed wild type levels of activity and, hence, interaction. This result suggests that residues Y124 and W127 of the N-CDA domain play critical roles in A3G oligomerization, whereas Y315 of the C-CDA does not.

### RNA-dependent oligomerization of A3G is mediated by the N-terminal CDA domain

**Figure 3:**
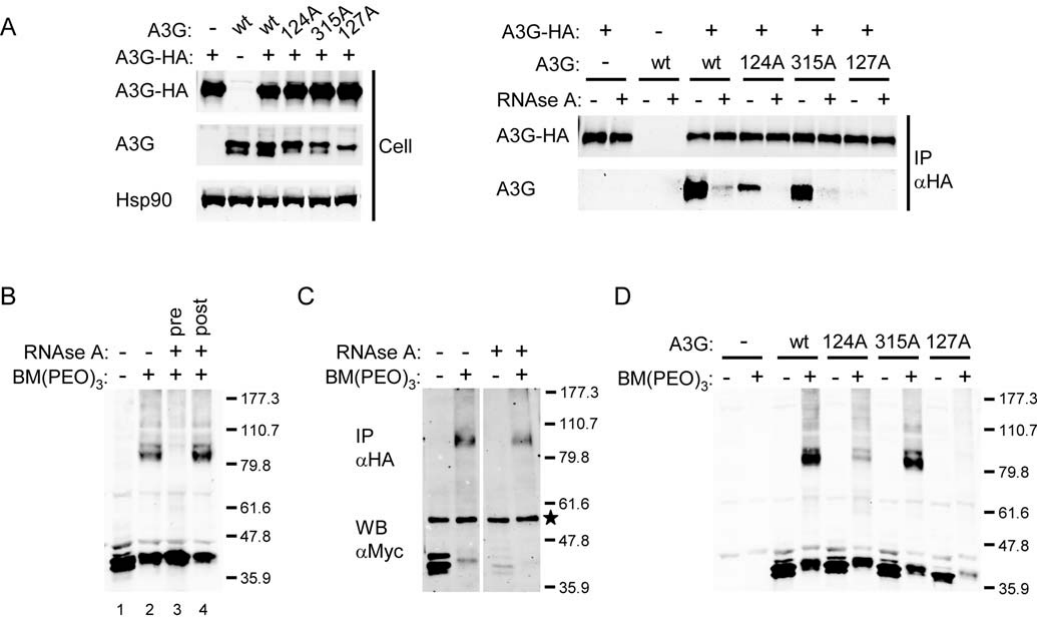
Oligomerization of wild type and mutant A3G proteins. (A) Co-immunoprecipitation of wild type and mutant A3G with HA-tagged wild type A3G (A3G-HA). Immunoblots on the left show whole cell expression of A3G, A3G-HA, and the cellular control protein Hsp90. On the right, blots show A3G and A3G-HA in the immunoprecipitate, with or without RNase A treatment. (B) Immunoblot showing A3G after chemical crosslinking with BM(PEO)_3_ in the lysate of transfected 293T cells. Lane 1, untreated control; lane 2, BM(PEO)_3_ treated; lane 3, BM(PEO)_3_ treated after incubation with RNase A; lane 4, BM(PEO)_3_ treated before incubation with RNase A. Relative molecular mass markers (in kD) are indicated on the right. (C) Immunoblot showing the immunoprecipitation of myc-tagged A3G (A3G-myc) with A3G-HA, with or without BM(PEO)_3_ and subsequent RNase A treatment. Samples were immunoprecipitated with anti-HA antibody, and immoblots were probed with the anti-myc antibody. An asterisk indicates the position of a band generated by crossreactivity to the heavy chain of the 3F10 antibody used for immunoprecipitation. (D) Immunoblot showing the effect of BM(PEO)_3_ treatment on wild type and mutant A3G in the lysates of transfected 293T cells. The control sample transfected with the empty vector is indicated by -.

To investigate further the oligomerization of A3G, we next performed a series of immunoprecipitation and chemical crosslinking experiments. Wild type or mutant A3G was co-expressed with HA-tagged wild type A3G (A3G-HA) and then co-immunoprecipitated from cell lysates using a monoclonal anti-HA antibody. Aliquots were or were not treated with RNAse A and analyzed by immunoblotting (Figure 3A). Consistent with the results of the yeast two-hybrid experiments, wild type A3G and the Y315A mutant were efficiently co-precipitated with A3G-HA, whereas co-precipitation of the Y124A mutant was strongly reduced and a mere trace amount of W127A was detected in the immunoprecipitate. In all cases, co-precipitation with A3G-HA was substantially inhibited by the treatment with RNAse A, indicating that RNA is required for stable A3G oligomerization.

We then performed a chemical crosslinking experiment in which cell lysates from 293T cells expressing wild type A3G were treated with BM(PEO)_3_, a compound with two reactive maleimide moieties separated by an 18 Å linker that form irreversible covalent bonds with the sulfohydrils of cysteines [36]. After crosslinking and resolution by SDS-PAGE, A3G was detected as a band migrating at ∼80 kD as well as at ∼40 kD where the untreated, and presumably monomeric, A3G migrates (Figure 3B, lanes 1 and 2). Treatment with RNase A prior to crosslinking resulted in complete disappearance of the band at ∼80 kD; this was maintained, however, when the RNase treatment was performed after the crosslinking reaction (Figure 3B, lanes 3 and 4, respectively). To verify that the crosslinked A3G species migrating at ∼80 kD represents an A3G oligomer, we also perfomed an experiment in which myc-tagged A3G (A3G-myc) was co-expressed with A3G-HA, subjected to crosslinking and then immunoprecipitated with the anti-HA antibody (Figure 3C). Samples were split into two aliquots which were (or were not) subjected to RNase A treatment after crosslinking. Indeed, A3G-myc was detected in the immunoprecipitate as a species migrating at ∼80 kD after treatment with BM(PEO)_3_ and this was unaffected by treatment with RNase A. In the control sample without the crosslinker, detection of monomeric A3G-myc in the immunoprecipitate was abolished by the treatment with RNase A. This result indicates that the species migrating at ∼80 kD is indeed formed by intermolecular crosslinking between A3G-HA and A3G-myc.

To assess in greater detail the oligomerization characteristics of some of our mutant A3G proteins, we next performed a crosslinking experiment with untagged wild type A3G or A3G harbouring the Y124A, Y315A or W127A mutations. The ∼80 kD crosslinked species appeared at a low level with the Y124A mutant and was barely detectable with the W127A mutant, whereas it was efficiently generated with the Y315A mutant (Figure 3D). Crosslinking and co-immunopreciptitaton of A3G with the Y124F or W127Y mutations resulted in increased levels oligomerization in comparison to the respective alanine mutations (Figure S1), which correlates with the less disruptive effects on virus packaging of these substitutions relative to the alanine mutations (Figure 1B and [35]). We note that we were unable to detect these subtle differences with the yeast-two hybrid system (Figure 1D).

To assess the possibility that the ∼80 kD species may be due to two A3G monomers being bound in close proximity on the same RNA molecule, we performed crosslinking experiments in which A3G was co-expressed with the RNA helicase Mov10. Mov10 is known to associate with A3G in ribonucleoprotein complexes in an RNA-dependent manner, though it is unknown whether these proteins interact directly [34]. We were unable to detect any intermolecular crosslinks between A3G and Mov10 (results not shown), further suggesting that the ∼80 kD crosslinked species forms as a consequence of A3G intermolecular contacts. Together, these results indicate that residues Y124 and W127 play central roles in the N-CDA mediated oligomerization of A3G, and that this interaction is also dependent on the presence of RNA.

### Modelling of an A3G dimer

**Figure 4:**
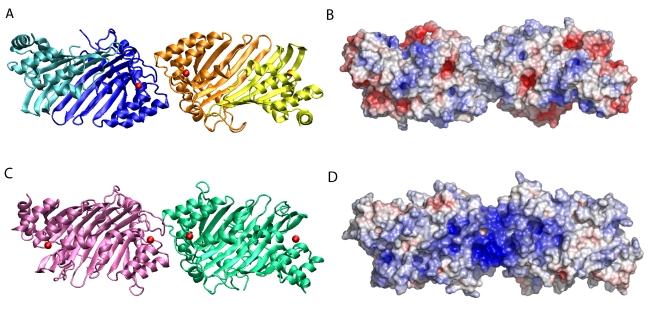
Structure of A2 and homology model of A3G. Ribbon representation of the A2 crystal structure (A) and the A3G homology model with the N-terminal CDA domain at the dimer interface (C). Monomer subunits of A2 are shown in turquoise, blue, orange, and yellow. Monomer subunits of A3G are shown in magenta and green. Zinc ions are shown as red spheres. To the right is a space-filling representation of the A2 crystal structure (B) and the A3G model (D) highlighting charge distribution. Red indicates negative charge and blue positive charge. The potential is ranged from −10*k*T (red) to the maximal positive value +10*k*T (blue).

In a complementary approach for addressing the mode of A3G oligomerization, we constructed homology models of A3G dimers using the A2 crystal structure as a template (Figure 4A). In these models, the N- and C-CDA domains of one A3G polypeptide together form the extended β-sheet that is the equivalent of an A2 dimer. Models with either the N-CDA or C-CDA at the oligomer interface, which corresponds to the tetramer interface of A2, were then assembled and subjected to energy minimization. In convergence with the results of the experiments described above, models with N-CDA at the oligomer interface (Figure 4C) proved energetically more favourable than models with the C-CDA at the dimer interface by ∼2000 to ∼3000 kJ/mol, depending on interactions with the solvent (Table S1). N-terminal dimerization resulted in residues Y124 and W127 being buried within the oligomer interface, with Y124 predicted to be slightly more accessible to solvent than W127 (Table S2). We next assessed the effect of the Y124A and W127A mutations by determining the interactions that are lost upon introduction of these mutations into the A3G structure model with the N-terminal CDA at the dimer interface (Table S3). This analysis demonstrated that the Y124A mutation results in the loss of intra- and intersubunit interactions, while the W127A mutation affects mostly intersubunit interactions in this structure model.

**Figure 5:**
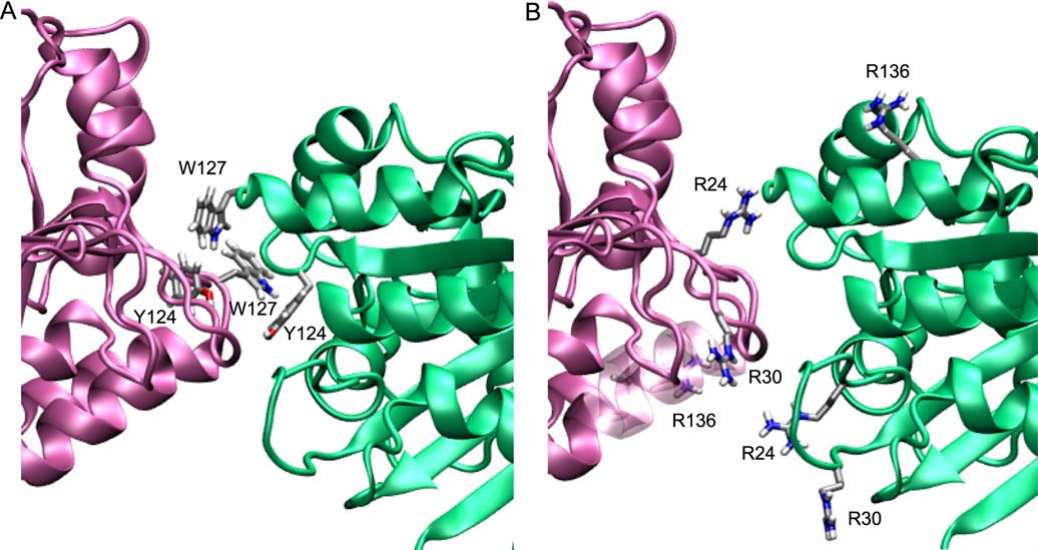
The dimer interface of the A3G homology model. (A) Detail of the predicted A3G dimer interface highlighting residues Y124 and W127 (A) and R24, R30, and R136 (B) from each subunit.

Importantly, inspection of the charge distribution over the surface the model revealed a conspicuous clustering of positive charges that are located in a rather large pocket at the predicted dimer interface (Figure 4D): notably, residues Y124 and W127 are also located within this pocket (Figure 5A). In contrast, this positively charged surface is absent from the A2 structure (Figure 4B). To assess the accuracy of this modelling effort, the C-CDA domain from our model was superimposed with the NMR [19] and X-ray [20] structures for this domain (Figure S2). The overall agreement was good with both structures, as evidenced by a root mean square deviation (RMSD) of less than 5 Å, but our model displayed slightly more similarity to the X-ray structure (RMSD NMR: 4.920 Å and RMSD X-ray: 3.650 Å).

### Mutational analysis of basic residues at the oligomer interface

**Figure 6:**
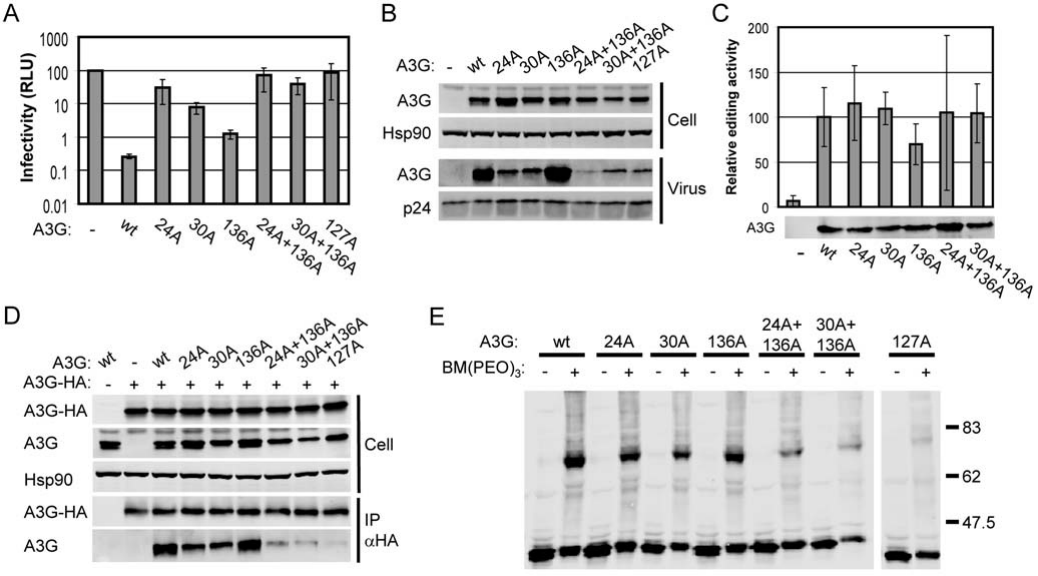
Characterization of A3G proteins with mutations at R24, R30, and R136. (A) Single-cycle infectivity of HIV-1/Δ*vif* viruses produced in the presence of wild type or mutant A3G. See the legend to Figure 2A. (B) Expression of wild type and mutant A3G proteins in 293T producer cells and packaging into HIV-1/Δ*vif* virions as determined by immunoblotting. (C) Relative editing activity of wild type and mutant A3G proteins as determined in a bacterial mutator assay relative to activity of wild type. See the legend to Figure 2C. (D) Co-immunoprecipitation of wild type and mutant A3G with HA-tagged wild type A3G (A3G-HA). Immunoblots on the top show whole cell expression of A3G, A3G-HA, and the cellular control protein Hsp90, as indicated by Cell at the left of the blot. At the bottom, blots show A3G and A3G-HA in the immunoprecipitate, as indicated by IP to the left of the blot. (E) Immunoblot showing the effect of BM(PEO)_3_ treatment on wild type and mutant A3G in the lysates of transfected 293T cells. The control reaction with the W127A was performed and blotted in parallel but was run on a separate gel.

The presence of clustered charged and aromatic residues at the A3G oligomer interface is suggestive of a binding site for RNA. In particular, the basic residues R14, R24, R29, R30, K63, K99, R102, R122, R136, K141 and R142 all lie within the aforementioned pocket and we sought to test this feature of our model. All these arginines and lysines were mutated to alanine and their antiviral properties assessed in single-cycle infectivity assays; the R24A and R30A proteins had the most profound loss of antiviral function, whereas mutations at other positions had no or modest effects, as exemplified by R136A (Figure 6A, and data not shown).

As the basic residues may act cooperatively to bind RNA, we also produced a set of doubly mutated proteins in which the R24A or R30A mutation was combined with alanine substitutions at R63, R99, R102, R136, K141 or R142. Only the R24A+R136A and R30A+R136A composite mutants showed further reductions in virus inhibition, resulting in phenotypes similar in severity to the W127A mutation (Figure 6A, and data not shown). Analysis of the levels of A3G present in virus particles revealed that mutants R24A, R30A, R24A+R136A and R30A+R136A were each packaged less efficiently than wild type A3G, but that the R136A mutant was still packaged well (Figure 6B). None of the R24A, R30A and R136A mutants showed any loss of editing activity in bacteria, either as single or double mutants, indicating that these proteins were not misfolded (Figure 6C).

We next assessed the oligomerization properties of these mutant A3G proteins by co-immunoprecipitation and chemical crosslinking. In both assay systems, we consistently observed that the R24A and R30A mutants oligomerized less efficiently than the wild type protein, and this was accentuated further by the additional R136A substitution (Figure 6D and 6E). Together, these results demonstrate that the removal of basic residues from the predicted oligomer interface creates proteins with very similar phenotypes to the Y124A and W127A mutants. Specifically, these mutated proteins display limited antiviral activity, packaging, and oligomerization, and this is consistent with the close spatial proximity of these residues in our structure model (Figure 5).

### Impaired A3G oligomerization correlates with reduced RNA association

**Figure 7:**
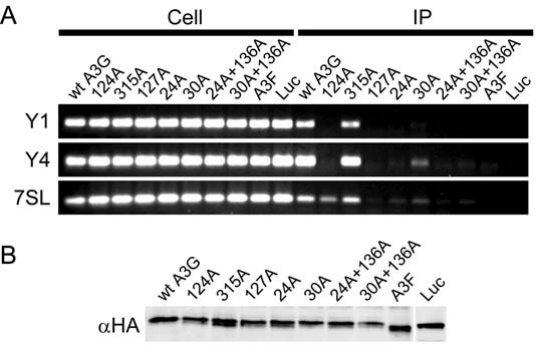
Association of wild type and mutant A3G with cellular RNAs. (A) Reverse transcription coupled PCR of RNA recovered from cell extracts and immunoprecipitates (indicated, respectively, by Cell and IP) of 293T cells transfected with wild type or mutant A3G-HA to detect selected cellular RNAs (Y1, Y4, and 7SL as indicated on the left). Negative controls were performed with HA-tagged A3F and luciferase (Luc). The control PCR reactions using immunoprecipitate with Taq polymerase instead of the RT enzyme were all negative and are not shown. (B) Immunoblot with anti-HA antibody showing the wild type and mutant A3G proteins present in the immunoprecipitate on which RT PCR was performed. Also included are A3F-HA and luciferase-HA (Luc) negative controls.

To determine whether oligomerization-defective mutants of A3G are reciprocally deficient for associating with cellular RNA, we performed semi-quantitative reverse transcription coupled PCR on immunoprecipitates of wild type or mutant HA-tagged A3G to detect the presence of the Y1, Y4 and 7SL RNAs; these RNA molecules have each previously been shown to be present in A3G-associated RNPs [13],[32],[37]. Indeed, these RNAs were readily detected in association with wild type A3G and the oligomer-forming Y315A mutant (Figure 7A). In contrast, much less Y1, Y4 and 7SL RNA was detected in the immunoprecipitates of W127A, R24A, R24A+R136A and R30A+136A A3G, as well as in the A3F and luciferase negative controls. Modest exceptions were the Y124A and R30A mutants, for which low levels of Y4 as well as 7SL RNA, respectively, were detected. These differences were not due to different amounts of protein in the immunoprecipitate, as demonstrated by immunoblotting (Figure 7B).

Thus, mutations of hydrophobic and basic residues at the predicted A3G oligomer interface caused a loss in association with cellular RNA. These mutations do not, however, affect the ability of A3G to assemble into high molecular weight ribonucleoprotein complexes in 293T cells, as evidenced by velocity sedimentation of A3G-containing cell lysates through sucrose gradients (Figure S3). Moreover, all A3G-containing complexes maintained sensitivity to RNase treatment, suggesting that the mutations have not imparted pleiotropic defects in nucleic acid interactions or the capability to assemble into large RNP complexes.

## Discussion

We have performed a mutational study of residues in the N- and C-CDA domains of A3G whose counterparts in A2 are involved in key interactions that support oligomerization of A2. Our results show that mutations in the N-CDA, but not the C-CDA, are associated with reductions in A3G RNA-dependent oligomerization and packaging into virions. Upon modelling of an A3G dimer onto the template A2 crystal structure, we identified a positively charged pocket at the oligomer interface formed between two N-CDAs that bore the hallmarks of a nucleic acid binding site (Figure 5). Indeed, mutation of basic residues within this pocket also resulted in losses of antiviral function, packaging into virus particles, oligomerization and association with cellular RNA (Figures 6 and 7). Consistent with previous work showing that only the C-CDA of A3G is responsible for DNA editing [5], [38]–[40], this attribute was unaffected by disruption of this basic pocket. Thus, our findings demonstrate further segregation of functions between the N- and C-CDA domains of A3G.

Throughout our chemical crosslinking experiments, we consistently observed the generation of oligomeric A3G migrating at ∼80 kD, which is twice the relative molecular mass of untreated A3G, which migrates at ∼40 kD (Figures 3 and 6). This result suggests that A3G oligomerizes as a discrete dimer, an assertion that is further supported by the fact that we did not detect slower migrating species at ∼120 (trimer) or ∼160 kD (tetramer). We note, however, that we have not formally demonstrated dimerization of A3G, as attempts to perform analytical ultracentrifugation were unsuccessful owing to the poor solubility of purified full-length A3G at high concentrations (results not shown). Nonetheless, dimerization of A3G via the N-terminal CDA domain remains the simplest model to explain our current results. Although this conclusion is at odds with a recent study proposing oligomerization of A3G via the C-CDA domain [41], that study is also contradicted by the observations that the C-CDA of A3G appears as a monomer by both ultracentrifugation [19] and crystallography [20].

Importantly, we have furthermore demonstrated that the oligomerizaton of A3G is dependent on the presence of RNA, as evidenced by the disruption of oligomers upon treatment with RNase (Figure 3). These observations are explained by our combined modelling and structure-function analyses, which predict that the oligomer interface between the A3G N-terminal CDA domains produces a positively charged pocket that requires occupation by RNA to allow effective oligomerization. Thus, we propose that the formation of A3G oligomers requires hydrophopic and basic residues that mediate protein-protein interactions between the A3G subunits and/or protein-RNA interactions, in a manner similar to that proposed for PKR and RIG-I [42]–[44]. We acknowledge, however, that the precise contribution of these residues to RNA-dependent oligomerization of A3G must await advances in the biochemical characterization of this protein.

An additional piece of evidence supporting the interdependence between the oligomerization of A3G and the association with RNA comes from the analysis of Y1, Y4 and 7SL RNA in A3G RNPs (Figure 7). In general terms, we observed that oligomerization-impaired mutants of A3G exhibited much reduced co-immunoprecipitation of these RNA molecules. Indeed, correlations between oligomer formation and RNA interaction were excellent in that the R30A and Y124A mutants displayed partial A3G-A3G interactions as well as intermediate levels of RNA interactions (Figures 3, 5, and 7). An additional instructive observation was made upon velocity sedimentation of cell lysates with oligomerization-impaired A3G, which demonstrated that assembly into RNase-sensitive high molecular weight RNP complexes was not noticeably affected by these mutations (Figure S3). This demonstrates that A3G's assembly into at least two intermolecular complexes is RNA-dependent: the oligomerization of A3G and its recruitment into RNP complexes. Importantly, our mutational analysis shows that oligomerization can be disrupted selectively without grossly preventing RNP association. This suggests either that (1) there is a certain degree of specificity to the identity of RNAs that are required for A3G oligomerization, but not to the RNAs that promote RNP association, or that (2) recruitment of A3G to RNase-sensitive RNP complexes is driven predominantly by protein-protein interactions. Specifically, RNA-dependent A3G RNP formation through protein-protein interactions could be mediated by proteins that bind A3G directly and additionally bind RNA.

Throughout these studies, we have highlighted a tight correlation between the packaging into HIV-1 virions and the RNA-dependent oligomerization of wild type and mutant A3G proteins. Here, we have presented a structure model of an A3G dimer that readily accommodates these attributes. Indeed, the packaging of A3G into virus particles has been reported to require binding to RNA and this has been interpreted as reflective of an RNA-dependent interaction between the HIV-1 Gag protein and A3G [10]–[14],[45]. Although the identity of RNA required for packaging of A3G into HIV-1 virions remains debated [10],[13],[14],[37],[45], specificity with regards to the RNA molecules that mediate oligomerization of A3G may impart some of the selectivity for the establishment of an A3G-Gag interaction and virion packaging.

The structure of A3G is also of considerable interest with regard to the binding of the HIV-1 Vif protein and efforts to manipulate this interaction therapeutically. Previous analyses have shown that Vif interacts with a three amino-acid core motif in A3G at residues 128–130 [35], [46]–[49], which is directly adjacent to residues Y124 and W127. This would position the residues of A3G that interact with Vif in close proximity to the oligomer interface. In our previous study, we found that mutant proteins with substitutions at position Y124 or W127 remain responsive to regulation by the Vif protein [35], suggesting that oligomerization is not a prerequisite for binding of Vif. Indeed, the interaction of Vif with A3G in co-immunoprecipitation experiments is resistant to treatment with RNase [30],[34]. Similarly, mutations at residues 128–130 in A3G affect the interaction with Vif but not packaging into virus particles [35] or generation of the dimeric species by chemical crosslinking (results not shown). Thus, while the residues that mediate Vif-binding and RNA-dependent oligomerization are in close proximity, they appear to be functionally distinct.

We have presented evidence for the RNA-dependent oligomerization of A3G via its N-CDA domain. A structure model of an A3G dimer based on the A2 crystal structure readily rationalizes the RNA-dependency of oligomerization as it revealed a clustering of positive charge near the predicted dimer interface. Furthermore, the model proved consistent with the contribution of basic residues at the interface to RNA-dependent oligomerization and packaging of A3G into virus particles. We thus propose that this model can serve as a guide for the further dissection of the structure-function relationships of domains and motifs within A3G. Ultimately, this may help endeavours aimed at therapeutic intervention with the interaction between the HIV-1 Vif protein and A3G. In particular, such efforts should strive to preserve the antiviral functions of A3G by interrupting the interaction with Vif, while maintaining the interactions that mediate association with RNA, oligomerization and virion packaging.

## Materials and Methods

### Plasmids and cloning

Wild type and mutant A3G expression plasmids for infectivity studies, immunoprecitation, crosslinking and the bacterial editing assay were generated as described previously [35]. A3G expression plasmids for the yeast two-hybrid experiments were generated by cloning of the EcoRI fragment from the pCMV4-A3G plasmids into the EcoRI site of the pGBKT7 (bait) and pHB18 plasmids (prey) [50]. Proper orientation and sequence of the insert was confirmed by restriction digest or sequencing.

### Single-cycle infectivity assays

Stocks of HIV-1/Δ*vif* [51] were prepared by cotransfection of 35-mm diameter monolayers of 293T cells with 0.5 µg of pA3G expression vector and 1.0 µg of pIIIB/Δ*vif* using polyethylenimine (PEI). After 24 hr, the supernatants were harvested and volumes corresponding to 5 ng p24^Gag^ used to infect 10^5^ TZM-bl indicator cells. The producer cells were lysed in SDS-containing loading dye for the analysis of protein expression. The induced expression of β-galactosidase in whole cell lysates was measured 24 hr after the initiation of infection using the Galacto-Star system (Applied Biosystems).

### Analysis of protein expression by immunoblotting

Whole cell lysates prepared from virus producing cells, immunoprecipitates and purified HIV-1 virions were resolved by SDS-polyacrylamide gel electrophoresis (SDS-PAGE, 11% gel) and analysed by immunoblotting using primary antibodies specific for A3G [5], myc (ab9106; Abcam), HA (12CA5), Hsp90 (sc7947: Santa Cruz) and p24^Gag^ [52]. Blots were resolved using either horseradish peroxidase-conjugated secondary antibodies and enhanced chemiluminescence (Pierce) or fluorescent secondary antibodies using the LI-COR infrared imaging technology (LI-COR UK LTD).

### Packaging assays

Virus stocks containing 20 ng p24^Gag^ were spun in a benchtop centrifuge at 21000× g for 2 h at 4°C through a 20% w/v sucrose cushion (500 µl) in a 2 ml eppendorf tube. Viral pellets were resuspended in loading dye and analyzed by immunoblotting. Whole cell lysates from the corresponding producer cells were assessed for A3G and Hsp90 expression in parallel.

### *E. coli* mutation assay

The KL16 strain of *E. coli* was transformed with pTrc99A-based, IPTG-inducible A3G expression vectors or the empty vector [53]. Individual colonies were picked and grown to saturation in LB medium containing 100 µg/ml ampicillin and 1 mM IPTG. Appropriate dilutions were spread onto agar plates containing either 100 µg/ml ampicillin or 100 µg/ml rifampicin and incubated overnight at 37°C. Mutation frequencies were recorded as the number of rifampicin-resistant colonies per 10^9^ viable cells, which were enumerated using the ampicillin-containing plates. Colony counts were recorded in this manner on 12 rifampicin- and 12 ampicilin-containing plates for each construct, in sets of 4 of each at one time. To average the repeat experiments, the average colony count for wild type A3G was set at 100 and all other scores were normalized to this value.

### Yeast two-hybrid assay

Yeast Y190 cells were transformed with 1 µg of each of the pGBKT7 (bait) and pHB18 (prey) plasmids [50]. The Wild type A3G cDNA was inserted into the bait construct, and Wild type A3G and mutant derivative inserts thereof were cloned into the prey construct. Transformants were selected on medium lacking tryptophan and leucine for 3 days at 30°C. Pools of >20 transformed yeast colonies were scraped into β-Gal assay buffer (60 mM Na_2_HPO_4_, 40 mM NaH_2_PO_4_, 10 mM KCl, 1 mM MgSO_4_, 50 mM 2-mercaptoethanol, 0.01% SDS, pH 7.0) and normalized according to optical density in a final volume of 500 µl. Cells were lysed by addition of 25 µl of chloroform and vortexing. The β-Gal substrate chlorophenol red-β-D-galactopyranoside was added to a final concentration of 4 mM and samples were incubated at room temperature for 30 min. After centrifugation to remove cellular debris, absorbance was determined at 540 nm. Repeat experiments were normalized to the OD_540_ of samples with Tsg101 (bait) and Vps28 (prey) which was set at 4.0.

### Coimmunoprecipitation assays

293T cells were transfected with 1 µg pA3G-HA and 1 µg pA3G (wild type or mutant) in 35-mm cultures. After 24 h, the cells were lysed in 600 µl lysis buffer (0.5% Triton X-100, 287 mM NaCl, 2.68 mM KCl, 1.47 mM KH_2_PO_4_, Na_2_HPO_4_, pH 7.2 and complete protease inhibitor cocktail from Roche). The lysates were cleared by centrifugation in a benchtop centrifuge at 21000× g for 10 min and 500 µl of each incubated with the 3F10 HA-specific antibody raised in rat (Roche) and protein G-agarose (Invitrogen) for 2 h at 4°C. 50 µl of the cleared lysate was kept to analyse protein expression levels. After binding to the beads the samples were washed twice with lysis buffer and split into two aliquots of 250 µl. To one aliquot of the samples 25 U of bovine pancreatic RNase A (Sigma) was added, and all samples were tumbled at room temperature for 30 min. The agarose beads were then washed three times with lysis buffer, and resuspended in 50 µl loading dye. 10 µl of the immunoprecipitated samples as well as 10 µl of the cleared lysate were resolved by SDS-PAGE (11% gel) and analyzed by immunoblotting using primary antibodies specific for HA, A3G or Hsp90.

### Chemical crosslinking

293T cells were transfected with 2 µg pA3G (wild type or mutant) in 35-mm cultures. After 24 h, the cells lysed in 600 µl lysis buffer (0.5% Triton X-100, 287 mM NaCl, 2.68 mM KCl, 1.47 mM KH_2_PO_4_, Na_2_HPO_4_, pH 7.2 and complete protease inhibitor cocktail from Roche). The lysates were cleared by centrifugation in a benchtop centrifuge at 21000× g for 10 min. Samples were then split into aliquots of 100 µl to which 10 U of RNase A was, or was not, added either prior to or after addition of 1.25 µl of 20 mM BM(PEO)_3_ (Thermo Scientific) in DMSO. After incubation at 20°C for 1 h, 1 µl of 1 M DTT was added to quench the reaction. After the addition of 25 µl loading dye, samples were analysed by SDS-PAGE and immunoblotting. In the experiment describing crosslinking of A3G-myc to A3G-HA, 293T cells were transfected with 2 µg of each plasmid. After 24 h, cells were lysed in 600 µl lysis buffer and cleared by centrifugation. Samples were split into aliquots of 250 µl which were treated, or not, with 2.5 µl of 20 mM BM(PEO)_3_ in DMSO. After addition of 2 µl 1 M DTT samples were incubated with the 3F10 anti-HA antibody (Roche) and protein A-agarose beads. Subsequent immunoprecipitation was performed in the manner described above and the gel resolved samples analysed using a myc-specific antibody.

### Reverse transcription coupled PCR

293T cells in a 10 cm dish were transfected with 12 µg of wild type or mutant A3G-HA expression vector and lysed after 24 h in lysis buffer (1% NP-40, 150 mM NaCl, 50 mM Tris-HCl pH 7.5 and complete protease inhibitor cocktail from Roche). The cell lysates were precleared overnight using an irrelevant monoclonal antibody and A3G ribonucleoprotein complexes were subjected to immunoprecipitation with the 3F10 rat anti-HA antibody using protein G-coupled agarose beads. Following immunoprecipitation, associated RNAs were recovered with the miRNAeasy mini kit (Qiagen). RNA was detected by semi-quantitative RT-PCR using the SuperScript III One-Step RT-PCR system with platinum Taq DNA polymerase (Invitrogen) (cDNA synthesis at 55°C for 30 min, denaturation at 95°C for 2 min, 15 amplification cycles of 95°C for 15 sec, 56°C for 30 sec and 68°C for 1 min, and a final extension step at 68°C for 5 min) using specific primers for Y and 7SL RNAs [37]. Products were resolved by electrophoresis on a 1.5% agarose gel and stained with ethidium bromide.

### Homology modelling of an A3G dimer

The structure of the dimer model of A3G was obtained by homology modelling using as a template the crystal structure of APOBEC2 (A2) (2NYT pdb entry). To generate the 3D-model, the alignment between A3G and APOBEC2 was submitted to the comparative structural modeling program MODELLER 8v2 [54]. 100 best solutions for the MODELLER objective function have been considered. Models were produced with either the N- or C-terminal CDA domain at the dimer interface.

### Velocity sedimentation

293T cells were transfected with 2 µg pA3G (wild type or mutant) in 35-mm cultures. After 24 h, the cells were lysed in 250 µl lysis buffer (0.626% NP40, 100 mM NaCl, 50 mM KAc, 10 mM EDTA, 10 mM Tris pH 7.4 and complete protease inhibitor cocktail from Roche). The lysates were cleared by centrifugation in a benchtop centrifuge at 162× g for 10 min followed by 18000× g for 30 sec. Samples were then split into aliquots of 100 µl to which 10 U of RNase A (Sigma) was, or was not, added. Samples were then loaded on top of a 10–15–20–30–50% sucrose step gradient in lysis buffer and centrifuged for 45 min at 163000× g at 4°C. After centrifugation, samples of 78 µl were sequentially removed from the top of the gradient, added to 30 µl of loading dye and analysed by immunoblotting.

## Supporting Information

Figure S1 Chemical crosslinking (A) and co-immunoprecipitation (B) of A3G proteins with the Y124A, Y124F, W127A, or W127Y mutations. Refer to the legend for Figure 3 for details.
(1.21 MB TIF)

Figure S2 Superposition of the C-CDA domain from the homology model of A3G (magenta) with the ten NMR models (blue, RMSD 4.920 Å) (A) and the crystal structure (yellow, RMSD 3.650 Å) (B)
(9.41 MB TIF)

Figure S3 Velocity sedimentation of wild type or mutant A3G through a sucrose gradient. The direction of the gradient is indicated at the top of the figure and treatment with RNase by a+at the right of the figure. Samples were examined by immunoblot using the A3G-specific antibody.
(2.03 MB TIF)

Table S1 Energy decomposition of N-terminal (N-N) and C-terminal (C-C) models for A3G oligomerization
(0.01 MB PDF)

Table S2 Solvent-accessible surface area (SASA) buried upon dimer formation as calculated with POPS
(0.05 MB PDF)

Table S3 Interactions lost upon introduction of the Y124A and W127A mutations into the structure model of the A3G dimer
(0.01 MB PDF)
