# Supplementary figures and images for: RNA-Dependent Oligomerization of APOBEC3G Is Required for Restriction of HIV-1
Source: PLoS Pathog. 2009 Mar 6;5(3):e1000330. doi: 10.1371/journal.ppat.1000330 (PMC2646141; doi:10.1371/journal.ppat.1000330)

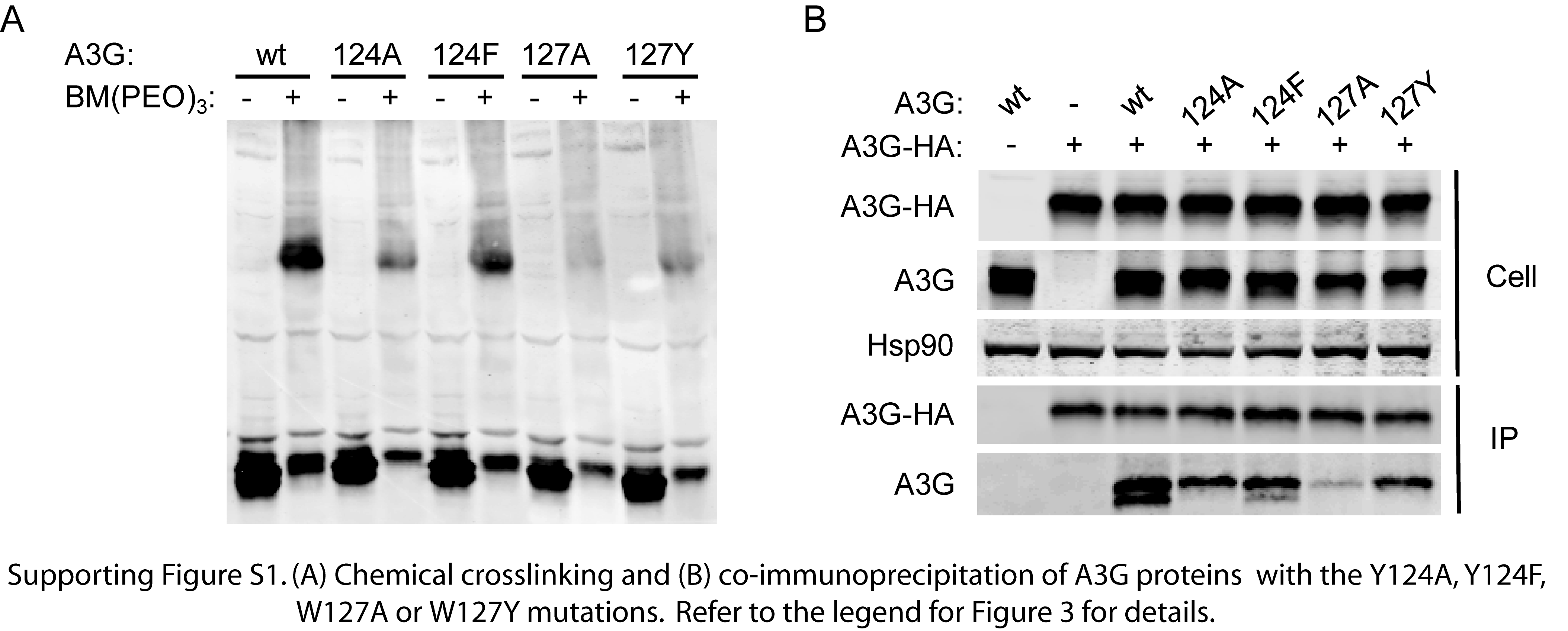

Supplement: Figure S1 — Chemical crosslinking (A) and co-immunoprecipitation (B) of A3G proteins with the Y124A, Y124F, W127A, or W127Y mutations. Refer to the legend for Figure 3 for details. (1.21 MB TIF) [file ppat.1000330.s001.tif]

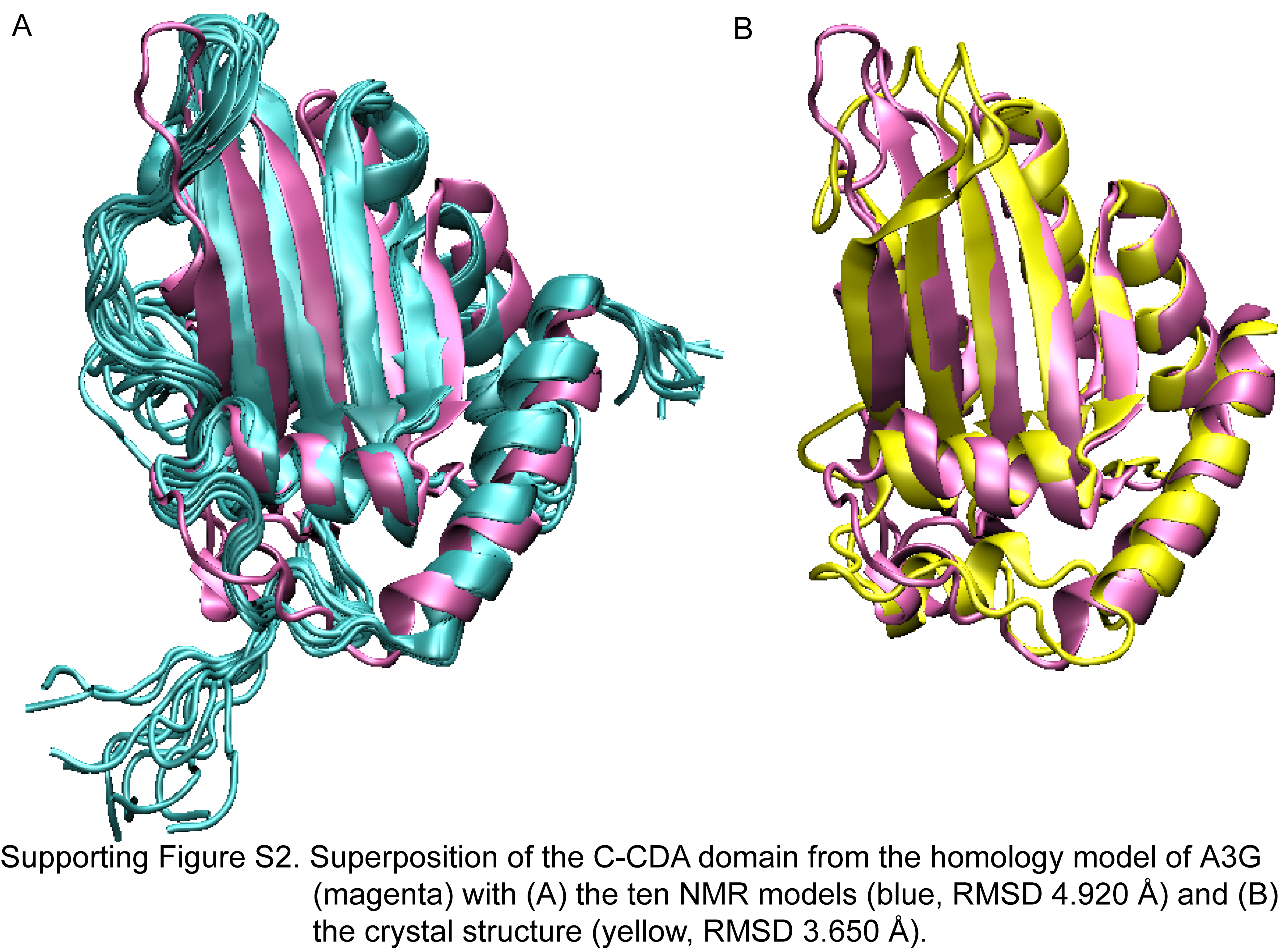

Supplement: Figure S2 — Superposition of the C-CDA domain from the homology model of A3G (magenta) with the ten NMR models (blue, RMSD 4.920 Å) (A) and the crystal structure (yellow, RMSD 3.650 Å) (B) (9.41 MB TIF) [file ppat.1000330.s002.tif]

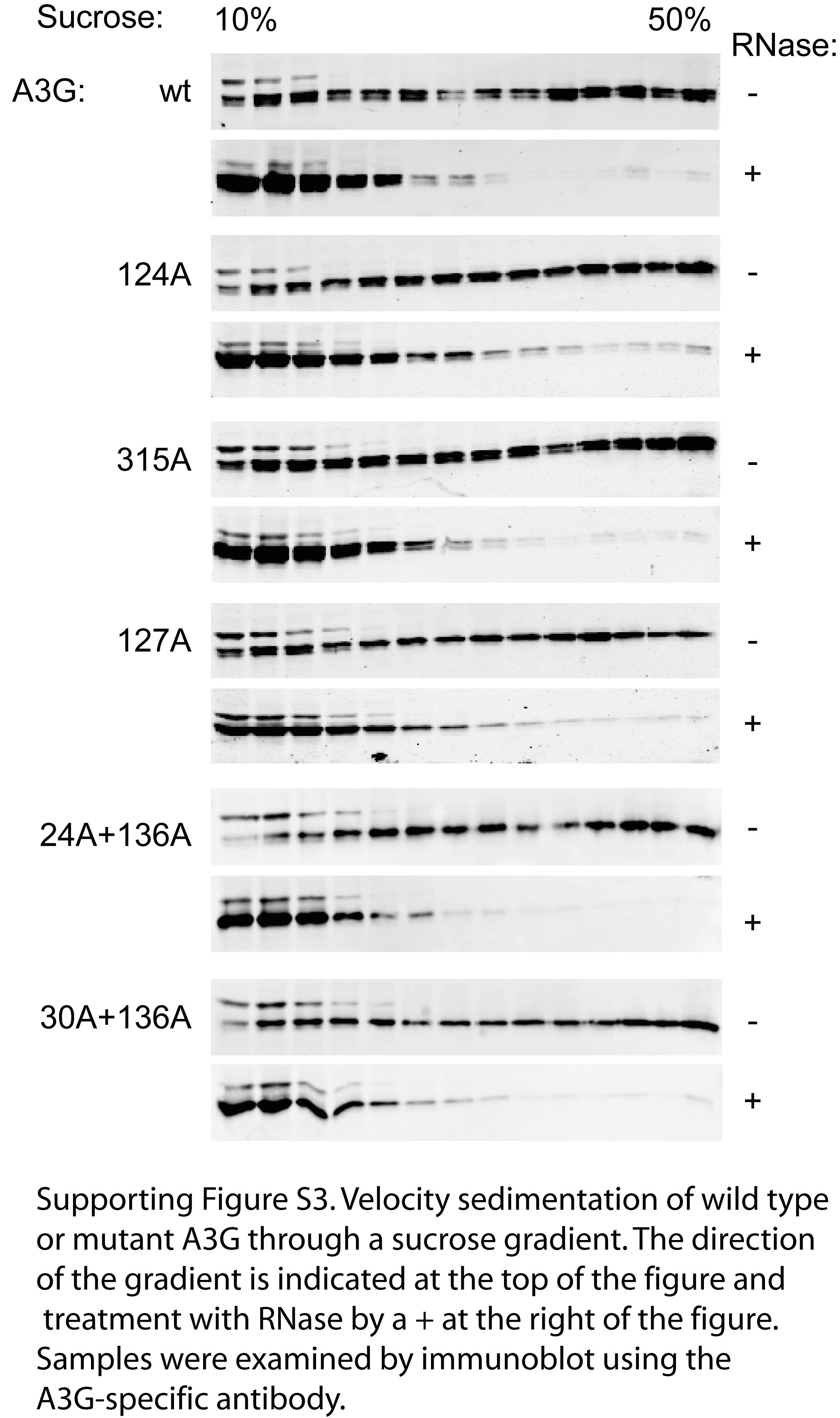

Supplement: Figure S3 — Velocity sedimentation of wild type or mutant A3G through a sucrose gradient. The direction of the gradient is indicated at the top of the figure and treatment with RNase by a+at the right of the figure. Samples were examined by immunoblot using the A3G-specific antibody. (2.03 MB TIF) [file ppat.1000330.s003.tif]
