# Supplementary material for: RNA-Dependent Oligomerization of APOBEC3G Is Required for Restriction of HIV-1
Source: PLoS Pathog. 2009 Mar 6;5(3):e1000330. doi: 10.1371/journal.ppat.1000330 (PMC2646141; doi:10.1371/journal.ppat.1000330)
Supplement: Table S1 — Energy decomposition of N-terminal (N-N) and C-terminal (C-C) models for A3G oligomerization (0.01 MB PDF) [file ppat.1000330.s004.pdf]

Energy kJ/mol	Model N-N	Model C-C
Coul:Dimer	-20447	-20004
LJ:Dimer	-28249	-27472
**Total Dimer**	**-48696**	**-45476**
Coul:Dimer-Solv	-43159	-41751
LJ:Dimer-Solv	-5244	-4999
**Total Dimer-Solv**	**-48403**	**-46750**

Supporting Table S1. Energy decomposition of N-terminal (N-N) and C-terminal (C-C) models for A3G oligomerization.
