# Supplementary material for: RNA-Dependent Oligomerization of APOBEC3G Is Required for Restriction of HIV-1
Source: PLoS Pathog. 2009 Mar 6;5(3):e1000330. doi: 10.1371/journal.ppat.1000330 (PMC2646141; doi:10.1371/journal.ppat.1000330)
Supplement: Table S2 — Solvent-accessible surface area (SASA) buried upon dimer formation as calculated with POPS (0.05 MB PDF) [file ppat.1000330.s005.pdf]

Residue	ΔSASA (Å^2)
**Monomer 1**	
R24	41.4
I26	15.4
L27	66.5
S28	32.5
W94	14.5
K99	28.6
R122	40.2
Y125	95.5
W127	107.5
D128	25.3
P129	16.7
**Monomer 2**	
Y22	26.2
L27	83.7
S28	40.7
R29	40.9
R122	28.4
Y124	23.6
W127	54.7
Y181	30.3

Supporting Table S2. Solvent accessible surface area (SASA) buried upon dimer formation as calculated with POPS**.**
