# Supplementary material for: RNA-Dependent Oligomerization of APOBEC3G Is Required for Restriction of HIV-1
Source: PLoS Pathog. 2009 Mar 6;5(3):e1000330. doi: 10.1371/journal.ppat.1000330 (PMC2646141; doi:10.1371/journal.ppat.1000330)
Supplement: Table S3 — Interactions lost upon introduction of the Y124A and W127A mutations into the structure model of the A3G dimer (0.01 MB PDF) [file ppat.1000330.s006.pdf]

	Y124A		W127A	
	**Monomer 1**	**Monomer 2**	**Monomer 1**	**Monomer 2**
**Monomer 1**	P25<br>I26<br>L27<br>P96	D128	M188	Y22<br>W94<br>Y124<br>Y125<br>F126
**Monomer 2**	Y125<br>W127<br>D128	P25<br>I26<br>L27<br>P96<br>C97	Y181	

Supporting Table S3. Interactions lost upon introduction of the Y124A and W127A mutations into the structure model of the A3G dimer.
